# Transcriptional activation of muscle atrophy promotes cardiac muscle remodeling during mammalian hibernation

**DOI:** 10.7717/peerj.2317

**Published:** 2016-08-11

**Authors:** Yichi Zhang, Oscar A. Aguilar, Kenneth B. Storey

**Affiliations:** Institute of Biochemistry and Department of Biology, Carleton University, Ottawa, ON, Canada

**Keywords:** *Ictidomys tridecemlineatus*, Muscle remodelling, Foxo, Western blotting, MAFbx, Ubiquitin ligase, Cardiac hypertrophy, Hibernation, MyoG, MuRF1

## Abstract

**Background.** Mammalian hibernation in thirteen-lined ground squirrels (*Ictidomys tridecemlineatus*) is characterized by dramatic changes on a physiological and molecular level. During hibernation, mammalian hearts show a propensity to hypertrophy due to the need for increasing contractility to pump colder and more viscous blood. While cardiac hypertrophy is quite often a process characterized by decompensation, the ground squirrel studied is an excellent model of cardiac plasticity and cardioprotection under conditions of hypothermia and ischemia. The forkhead box O (Foxo) family of proteins and myogenin (MyoG) are transcription factors that control protein degradation and muscle atrophy by regulating the expression of the E3 ubiquitin ligases, MAFbx and MuRF1. These ligases are part of the ubiquitin proteasome system by transferring ubiquitin to proteins and targeting these proteins for degradation. Regulation of Foxo1 and 3a occurs through phosphorylation at different residues. The threonine-24 (Thr-24) and serine-319 (Ser-319) residues on Foxo1, and the Thr-32 residue on Foxo3a are phosphorylated by Akt, leading to cytoplasmic localization of Foxo. We propose that the described mechanism contributes to the changes taking place in cardiac muscle throughout hibernation.

**Methods.** Total and phosphorylated protein levels of Foxo1 and Foxo3a, as well as total protein levels of MyoG, MAFbx, and MuRF1, were studied using immunoblotting.

**Results.** Immunoblotting results demonstrated upregulations in Foxo1 and Foxo3a total protein levels (1.3- and 4.5-fold increases relative to euthermic control, for Foxo1 and 3a respectively) during late torpor, and protein levels remained elevated throughout the rest of torpor and at interbout arousal. We also observed decreases in inactive, phosphorylated Foxo1 and 3a proteins during throughout torpor, where levels of p-Foxo1 Ser^319^ and Thr^24^, as well as p-Foxo3a Thr^32^ decreased by at least 45% throughout torpor. MyoG was upregulated only during late torpor by 2.4-fold. Protein levels of MAFbx and MuRF1 increased in late torpor as well as during early arousal by as much as 2.8-fold, and MAFbx levels remained elevated during interbout arousal, whereas MuRF1 levels returned to control levels.

**Discussion.** The present results indicate that upregulation and activation of Foxo1 and 3a, in addition to the increase in MyoG levels at late torpor, may be upregulating the expression of MAFbx and MuRF1. These findings suggest that there is activation of the ubiquitin proteasome system (UPS) as ground squirrels arouse from torpor. Therefore, the signalling pathway involving MyoG, and the E3 ligases MAFbx and MuRF1, plays a significant role in cardiac muscle remodelling during hibernation. These findings provide insights into the regulation of protein degradation and turnover in the cardiac muscle of a hibernator model.

## Introduction

Physiological adaptation to environmental changes is vital to the survival of many organisms. In order to survive prolonged seasonal exposure to food scarcity and frigid temperatures, some mammals that reside in northern climates hibernate during the winter. The thirteen-lined ground squirrel (*Ictidomys tridecemlineatus*) is one example of a deep hibernator. During the hibernation season, this animal undergoes periods of deep torpor where its metabolic rate is depressed (often to just 2–4% of normal conditions) and its body temperature (*T*_*b*_) decreases from 37 °C to near ambient temperatures (0–5 °C) (Frerichs & Hallenbeck, 1998). These periods of torpor (often 1–2 weeks or more) are interspersed with brief periods of arousal, where metabolic rate returns to euthermic levels and *T*_*b*_ rises to 37 °C. Metabolic rate depression saves hibernating ground squirrels up to 88% of euthermic ATP expenditure, ATP that they then redistribute for use in essential cell functions (Morin & Storey, 2007; Storey, 2010; Wang & Lee, 2011).

Each organ/tissue of the hibernator must make specific adjustments at low *T*_*b*_ to support long term torpor. Heart rate is strongly reduced during torpor, often from euthermic rates of 350–400 beats/min to just 5–10 beats/min. These changes in heart rate, and the increased viscosity of blood at low *T*_*b*_ values, requires a reorganization in heart dynamics (Frerichs et al., 1994; Frerichs & Hallenbeck, 1998). The strength of each individual contraction must be significantly increased in order to pump colder, and more viscous blood through the hibernator’s body; as a result cardiac hypertrophy is observed (Zhou et al., 1991; Wickler, Hoyt & Van Breukelen, 1991; Depre et al., 2006; Nakipova et al., 2007). There are two types of cardiac hypertrophy with one being exercise-induced (physiological hypertrophy) and the other being induced by hemodynamic loading, neurohumoral activation, or other stresses on the heart (pathological hypertrophy) (Hill & Olson, 2008). Prolonged pathological hypertrophy leads to decompensation; resulting in significant cardiac fibrosis, which is characterized by collagen deposition and stiffening of cardiac chamber walls. This results in a reduction of diastolic filling, which ultimately prevents the heart from pumping enough blood to meet body demands; this is a condition known as heart failure (Hill & Olson, 2008; Day, 2013). As mentioned previously, hibernating ground squirrels naturally undergo an adaptive form of cardiac hypertrophy (Wickler, Hoyt & Van Breukelen, 1991; Nelson et al., 2008; Nelson & Rourke, 2013; Bogren et al., 2014). This fascinating process is orchestrated by molecular adaptations that occur during hibernation, which are just beginning to be discovered (Morano et al., 1992; Nakao et al., 1997; Kinugawa et al., 2001; Brauch et al., 2005; Tessier & Storey, 2012; Luu et al., 2015; Zhang & Storey, 2015). Understanding the mechanism of adaptive cardiac hypertrophy in hibernators could provide novel insight into the prevention and potential treatment of maladaptive cardiac hypertrophy and heart failure.

One of the key elements of cardiac hypertrophy is the stress-induced adaptation in protein turnover; which involves protein synthesis and degradation. Both of these mechanisms are activated by increased cardiac workload due to pressure or volume (Depre et al., 2006). Upregulation of Nuclear Factors of Activated T Cells (NFAT) and Myocyte Enhancer Factor-2 (MEF2) have been implicated in the increased synthesis of numerous proteins during cardiac hypertrophy (Tessier & Storey, 2012; Zhang & Storey, 2015; Zhang & Storey, 2016). In the postnatal myocardium, these transcription factors represent part of the fetal gene expression profile, which controls pathological cardiac hypertrophy and the development of heart failure (Frey & Olson, 2003; Frey et al., 2004). We suspect that molecular pathways promoting protein degradation may play a role at various stages of hibernation in order to counteract the fetal gene expression profile and avoid pathological hypertrophy. The ubiquitin proteasome system (UPS) is one such mechanism of protein degradation, whereby substrates are ligated to ubiquitin via ubiquitin ligases and are targeted for degradation (Herrmann, Lerman & Lerman, 2007). The specificity of the UPS is determined by E3 ubiquitin ligases that recognize specific target proteins; E3 ligases include Muscle Atrophy F-Box (MAFbx/atrogin-1) and Muscle Ring Finger (MuRF). These E3 ligases transfer ubiquitin to the amino group of a lysine residue on the target protein in order to target the substrate for degradation (Dantuma & Lindsten, 2010). The importance of MAFbx and MuRF1 in relation to skeletal muscle atrophy has been demonstrated by studies which showed significantly lower losses in muscle mass in MAFbx/MuRF1-null mice (MAFbx -/-; MuRF1 -/-), and other lines of evidence that show a correlation between atrophy and MAFbx/MuRF1 upregulation (Bodine et al., 2001; Foletta et al., 2011; Bodine & Baehr, 2014). The role of the UPS remains relatively unclear in cardiac muscle; although MuRF1-null mice showed resistance to dexamethasone-induced cardiac atrophy and resistance to atrophy following the reversal of transaortic constriction (TAC), which induced cardiac hypertrophy in the mice. Therefore, following reversal of TAC, the hearts of MuRF -/- mice remained enlarged (Willis et al., 2009). Also, MAFbx and MuRF1 have shown upregulation in association with cardiac hypertrophy or heart failure (Depre et al., 2006; Galasso et al., 2010). This increase in expression of ubiquitination machinery may be in response to the protein overproduction that accompanies hypertrophy or in response to modified or damaged proteins that need to be degraded (Day, 2013).

**Figure 1:**
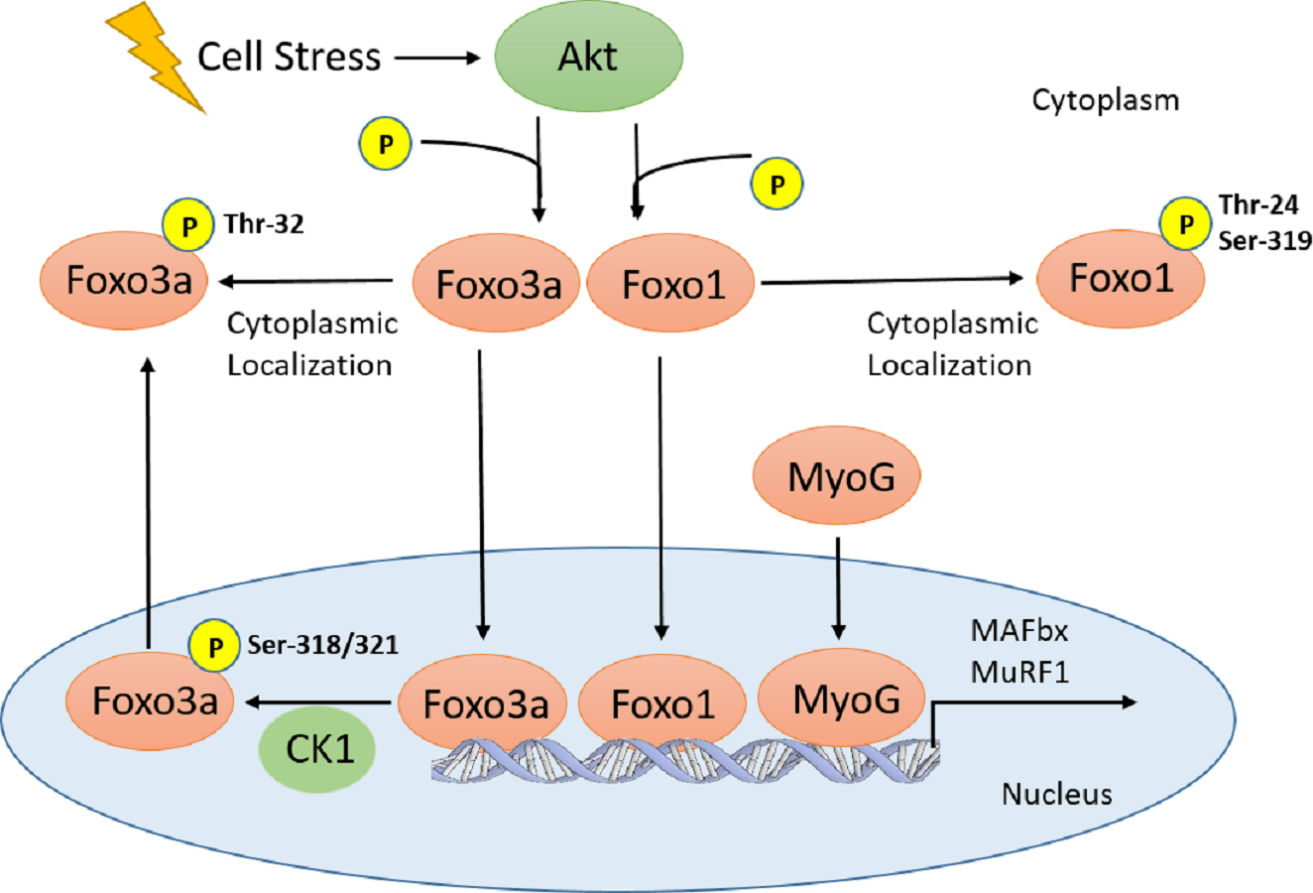
Schematic of Akt regulation of Foxo1 and Foxo3a activity. Cellular stresses (i.e., hyperoxia, increase in *T*_*b*_) activate Akt, which inhibits Foxo translocation to the nucleus through phosphorylation on several residues for Foxo1 and 3a. In the nucleus, Foxo3a phosphorylation at Ser-318, 321 by CK1 results in nuclear exclusion. Therefore, all Foxo1 and 3a phosphorylation sites are inhibitory. This signaling pathway, along with MyoG, is involved in E3 ubiquitin ligase (MAFbx and MuRF1) regulation which result in physiological outcomes such as muscle remodelling and protein degradation/atrophy in skeletal and heart muscle.

Early studies have shown that both MAFbx and MuRF1 are upregulated under similar atrophy-inducing conditions, suggesting that both ligases are regulated by common transcription factors; Foxos were the first set of such factors to be identified (Sandri et al., 2004; Stitt et al., 2004). For example, Foxo1 was shown to increase MAFbx or MuRF1 levels by blocking their inhibition from the IGF-1/PI3K/Akt insulin signalling pathway. Thus, it was initially believed that Foxo1 indirectly increases the expression of MAFbx and MuRF1 (Stitt et al., 2004). However, later studies identified that Foxo transcription factors share consensus sequences that allow them to bind directly to the MAFbx and MuRF1 promoters (Sandri et al., 2004; Waddell et al., 2008). Akt, otherwise known as protein kinase B (PKB), has been shown to block the function of all three Foxo proteins through phosphorylation, leading to their containment in the cytoplasm (Brunet et al., 1999; Takaishi et al., 1999; Tang et al., 1999). Conversely. dephosphorylation of Foxo factors leads to nuclear localization and upregulation of genes that promote growth suppression, apoptosis, reactive oxygen species (ROS) response, and muscle atrophy (Takaishi et al., 1999; Essers et al., 2004; Dobson et al., 2011; Wu & Storey, 2014). Akt has numerous phosphorylation sites on Foxo1 and 3a, including Threonine^32^ (Thr^32^) for Foxo3a, as well as Thr^24^ and Serine^319^ (Ser^319^) for Foxo1 (Nakae, Barr & Accili, 2000; Cahill et al., 2001; Rena et al., 2001; Dobson et al., 2011) (Fig. 1). Aside from Akt, Foxos can also be phosphorylated and inhibited by numerous other kinases; including Jun N-terminal kinase (JNK), AMP-activated protein kinase (AMPK), cyclin-dependent kinase (CDK), and MAPK-activated protein kinase (MK) (Huang et al., 2006; Van der Horst & Burgering, 2007; Yuan et al., 2008; Kress et al., 2011) (Fig. 1).

Other than the Foxo transcription factors, myogenin (MyoG) has also been identified as a positive regulator of MAFbx and MuRF1 (Fig. 1); the expression of both ligases as well as muscle atrophy were attenuated in MyoG-null mice (Moresi et al., 2010). Given the natural tendencies of hibernators to undergo adaptive cardiac hypertrophy over the course of the hibernation season, cardiac remodelling involving protein turnover is believed to occur. In the thirteen-lined ground squirrel, various heart proteomics and transcriptomics studies have been performed (Hampton et al., 2011; Vermillion et al., 2015a; Vermillion et al., 2015b). Two transcriptomics studies have characterized the gene expression of Foxo1, Foxo3, MyoG, MAFbx, and MuRF1 (Hampton et al., 2011; Vermillion et al., 2015a). However, there has not been a study that has characterized the protein expression of these targets related to muscle atrophy. Therefore, the present study analyzed the protein expression of Foxo1 and 3a, along with their various phosphorylated forms, in addition to MyoG, MAFbx, and MuRF1 over torpor-arousal cycles in the cardiac muscle of thirteen-lined ground squirrels. We hypothesize that the Foxo and MyoG transcription factors in addition to the E3 ligases MAFbx and MuRF1 would play a significant role in this process.

## Materials and Methods

### Animal treatment

Thirteen-lined ground squirrels (*I. tridecemlineatus*), weighing 150–300 g, were captured and transported by USDA licensed personnel (TLS Research, Bloomingdale, IL, USA). Animals experiments were conducted at the Animal Hibernation Facility, National Institute of Neurological Disorders and Stroke, NIH, Bethesda, MD where experiments were conducted by the laboratory of Dr. JM Hallenbeck as previously described (McMullen & Hallenbeck, 2010). All animal procedures were approved by the Animal Care and Use Committee of the National Institute of Neurological Disorders and Stroke (NIH; animal protocol no. ASP 1223-05). Squirrels from both genders were sampled equally with a mixture of genders for each condition, and all animals were 1–3 years of age, although the exact age of each animal was unknown since animals were wild-captured. Animals were fitted with a sterile programmable temperature transponder (IPTT-300; Bio Medic Data Systems) to monitor *T*_*b*_. Squirrels were anaesthetized with 5% isofluorane and the transponder was injected subcutaneously into the intrascapular region. Torpor was induced in the animals by maintaining constant darkness in an environmental chamber at 4–5 °C. All animals had been through several torpor-arousal bouts prior to sampling at the randomly selected stage of the torpor-arousal stage. Four animals were euthanized and hearts were immediately excised and frozen in liquid nitrogen. Tissue samples were subsequently stored at −80 °C until use. This procedure was repeated for each of the six different time points defined by *T*_*b*_, duration of torpor, and respiration rate, which were: (1) Euthermic Cold room (EC); these squirrels had a stable *T*_*b*_ of 34–37 °C for at least three days and were capable of entering torpor but had not done so in the past 72 h. (2) Early entrance into torpor (EN); *T*_*b*_ falling with sampling between 31 °and 18 °C. (3) Early torpor (ET); *T*_*b*_ stable at ∼5–8 °C for <24 h. (4) Late torpor (LT); *T*_*b*_ stable at ∼5–8 °C for >5 days. (5) Early Arousal (EA); *T*_*b*_ rising to at least ∼12 °C with a rapid increase in respiration to ≥60 breaths/min, (6) Interbout arousal (IA)—where the animals were naturally aroused after the torpor phase of the hibernation bout and reached the respiratory rate, metabolic rate, and body temperature of fully aroused animals for 6 h after being in torpor for at least 5 days. These animals remain in the hibernaculum (4 °C) but their core body temperature is back to ∼37 °C. A schematic representation of the torpor-arousal cycle is shown in a previous review written by Tessier & Storey (2016).

### Total protein extraction

Samples of frozen heart from *n* = 4 individuals from each of the six time points throughout the torpor-arousal cycle (EC, EN, ET, LT, EA, IA) were extracted; aliquots of ∼0.5 g tissue were quickly weighed, powdered into very small pieces under liquid nitrogen before homogenization using a Polytron PT10 in 1:3 w:v ice-cold homogenizing buffer (20 mM Hepes, 200 mM NaCl, 0.1 mM EDTA, 10 mM NaF, 1 mM Na_3_VO_4_, 10 mM *β*-glycerophosphate) with 1 mM phenylmethylsulfonyl fluoride (Bioshop) and 1 µL/mL protease inhibitor cocktail (Bioshop) added immediately before homogenization. Samples were then centrifuged at 10,000 rpm (10 min, 4 °Cgree C), after which the supernatant was removed. Protein concentration was measured via the Coomassie blue dye-binding method using the BioRad reagent (BioRad Laboratories, Hercules, CA, USA) at 595 nm on a MR5000 microplate reader. Samples were then adjusted to a constant of 10 µg/µL by addition of small amounts of homogenizing buffer and then aliquots were combined 1:1 v:v with 2x SDS loading buffer (100 mM Tris-base, pH 6.8, 4% w:v SDS, 20% v:v glycerol, 0.2% w:v bromophenol blue, 10% v:v 2-mercaptoethanol) and then boiled. The final protein samples (5 µg/µL) were stored at −20 °C until use.

### Western blotting

Sample aliquots containing 25 µg of protein were loaded onto 8% [Foxo1, 3a, 4, phosphorylated-Foxo1 (T24), p-Foxo3a (T32), p-Foxo1 (S319), p-Foxo3a (S318/321), MuRF1] or 10% (MyoG, MAFbx) polyacrylamide gels and were run at 180 V for 60 min. Proteins were then wet transferred to PVDF membranes by electroblotting at 160 mA for 1.5 h using a transfer buffer containing 25 mM Tris (pH 8.5), 192 mM glycine and 10% v:v methanol at room temperature. Membranes were washed with 1x TBST (20 mM Tris base, pH 7.6, 140 mM NaCl, 0.05% v:v Tween-20 in ddH_2_O) for 3 × 5 min. Membranes were then blocked for 30 min with 5% (for Foxo1, 3a, p-Foxo1, p-Foxo3a) or 7.5% (MyoG, MAFbx, MuRF1) w:v milk in 1x TBST. After washing again for 3 × 5 min with 1x TBST, membranes were probed with specific primary antibodies at 4 °C overnight. Primary antibodies used were: rabbit polyclonal MyoG (sc-576), MAFbx/atrogin-1 (sc-27645), MuRF1 (gtx110475), Foxo1 (gtx110724), Foxo3a (gtx100277), p-Foxo1/FKHR Ser^319^ (Genescript A00373), p-Foxo3a Ser^318/321^ (cs 9465), and p-Foxo1 Thr^24^/p-Foxo3a Thr^32^ (cs 9464P). All antibodies were used at a 1:1,000 v:v dilution in 1x TBST. After probing with primary antibody, membranes were washed for 3 × 5 min with 1x TBST and then incubated with HRP-linked anti-rabbit IgG secondary antibody (Bioshop; 1:6,000 v:v dilution) for 30 min at room temperature. Bands were visualized by enhanced chemiluminescence (H_2_O_2_ and Luminol) and then blots were restained using Coomassie blue to quantify and visualize total protein. Immunoblot bands for ground squirrel proteins corresponded to the molecular weights indicated on the antibody specification sheets from respective manufacturers, as confirmed by running PINK Plus Prestained Protein Ladder (FroggaBio).

### Quantification and statistics

Band densities on chemiluminescent immunoblots were visualized using a Chemi-Genius BioImaging system (Syngene, Frederick, MD) and quantified using the Gene Tools software. Immunoblot band density in each lane was standardized against the summed intensity of a group of Coomassie-stained protein bands in the same lane; this group of bands was chosen because they were not located close to the protein band of interest but were prominent and constant across all samples. This method of standardizing against a total protein loading control has been suggested to be more accurate in comparison with standardizing against housekeeping proteins such as tubulin (Eaton et al., 2013). Data are expressed as means ±SEM, *n* = 4 independent samples from different animals. Statistical testing used the one-way ANOVA and the Tukey post-hoc functions from the GraphPad Prism software (San Diego, CA).

## Results

### Analysis of Foxo1 and p-Foxo1 protein levels

**Figure 2:**
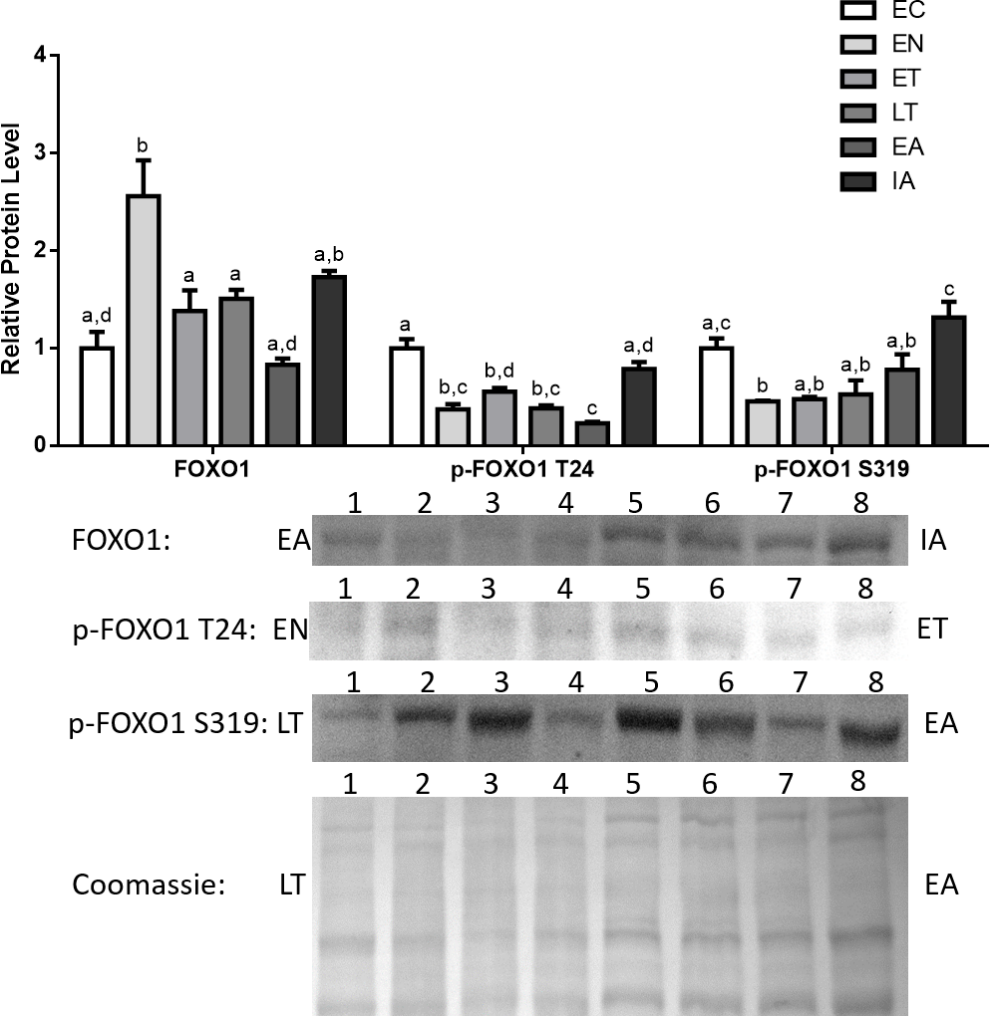
Changes in the protein levels of the Foxo1 transcription factor and its phosphorylated forms Ser^319^ (S319) and Thr^24^ (T24) over the course of the torpor-arousal cycle in cardiac muscle of *I. tridecemlineatus*. Foxo1, p-Foxo1 S319, and T24 protein expression levels were visualized at six sampling points: euthermic in the cold room (EC), entrance into torpor (EN), early torpor (ET), late torpor (LT), early arousal (EA), and interbout arousal (IA). See ‘Materials and Methods’ for more extensive definitions. Representative immunoblots and Coomassie total protein loading controls are shown for selected pairs of sampling points that are labeled on the left and right of the gel. Sample numbers (lanes) are labeled along the top indicating an *n* = 4 of one sampling point and an *n* = 4 of the other sampling point (i.e., for Foxo1: EA lanes 1, 2, 3, 4 and IA lanes 5, 6, 7, 8). Histograms show mean band densitometries (±S.E.M., *n* = 4 independent protein isolations from different animals). Data were analyzed using analysis of variance with a post hoc Tukey’s test (*p* < 0.05); for each parameter measured, values that are not statistically different from each other share the same letter notation.

Total Foxo1, p-Foxo1 (Thr^24^), and p-Foxo1 (Ser^319^) protein levels in cardiac muscle were analyzed by immunoblotting that compared animals sampled from six different stages of the torpor-arousal cycle: EC, EN, ET, LT, EA, IA (Fig. 2). Total Foxo1 levels immediately rose by 2.3-fold in comparison with euthermic control (EC) upon entering torpor (EN) and declined during torpor (ET, LT). However, Foxo1 levels were still elevated (1.6- and 1.3-fold relative to EC, for ET and LT respectively) before decreasing during arousal (Fig. 2). In contrast, protein levels for phosphorylated Foxo1 at Thr^24^ (p-Foxo1 T24), one of the inhibitory phosphorylation sites targeted by Akt (Dobson et al., 2011), declined and remained low throughout the torpor-arousal cycle and returned to euthermic levels during IA (Fig. 2). Ser^319^ (S319) is another inhibitory phosphorylation site on Foxo1 that is targeted by Akt (Dobson et al., 2011), p-Foxo1 S319 levels dropped upon entry and during torpor (EN, ET, LT) by 47–54% in comparison with EC.

### Analysis of Foxo3a and p-Foxo3a protein levels

**Figure 3:**
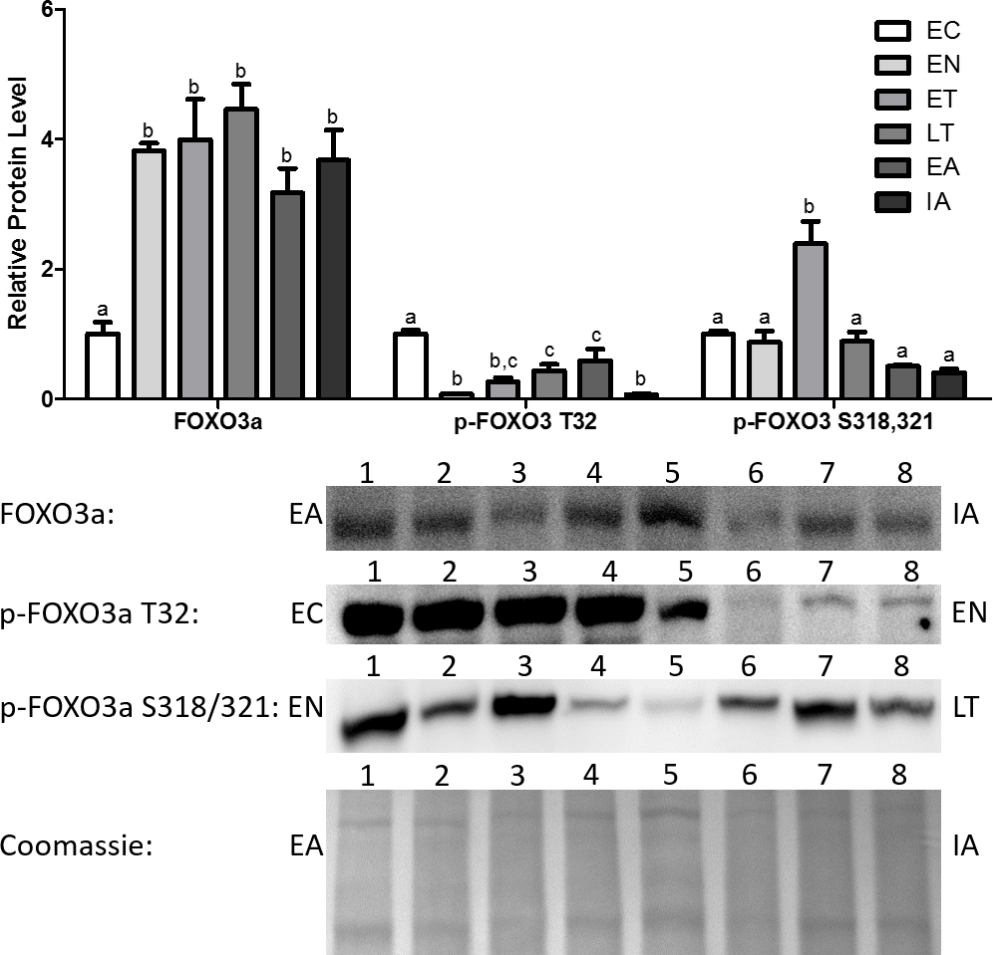
Changes in protein levels of the Foxo3a transcription factor and its phosphorylated forms Ser^318/321^ (S318/321) and Thr^32^ (T32) over the course of the torpor-arousal cycle in the cardiac muscle of *I. tridecemlineatus*. Representative immunoblots and Coomassie total protein loading controls are shown for selected pairs of sampling points that are labeled on the left and right of the gel. Sample numbers (lanes) are labeled along the top indicating an *n* = 4 of one sampling point and an *n* = 4 of the other sampling point (i.e., for Foxo3a: EA lanes 1, 2, 3, 4 and IA lanes 5, 6, 7, 8). Histograms show mean band densitometries (±S.E.M., *n* = 4 independent protein isolations from different animals). Data were analyzed using analysis of variance with a post hoc Tukey’s test (*p* < 0.05); for each parameter measured, values that are not statistically different from each other share the same letter notation.

Total Foxo3a levels increased dramatically upon entering torpor and remained elevated throughout the torpor-arousal cycle, with the peak occurring at LT (4.5-fold in comparison with EC) (Fig. 3). Akt is known to inhibit Foxo3a from translocating to the nucleus and regulating the transcription of genes by phosphorylating Foxo3a at T32 (Dobson et al., 2011). p-Foxo3a T32 protein levels decreased dramatically by 92% relative to EC at EN, and although protein levels rose throughout torpor and during early arousal (EA), these levels were still significantly decreased in comparison with EC (Fig. 3). p-Foxo3a S318,321 targets phosphorylation sites that are unrelated to the phosphorylation sites targeted by common Foxo3a inhibitory proteins like Akt (Fig. 1) (Rena et al., 2002; Dobson et al., 2011). p-Foxo3a S318, 321 protein levels remained steady throughout most of the torpor-arousal cycle, but it increased dramatically during ET (2.4-fold increase relative to EC), contrasting the trend observed with the other p-Foxo proteins.

### Analysis of MyoG, MAFbx, and MuRF1 protein levels

**Figure 4:**
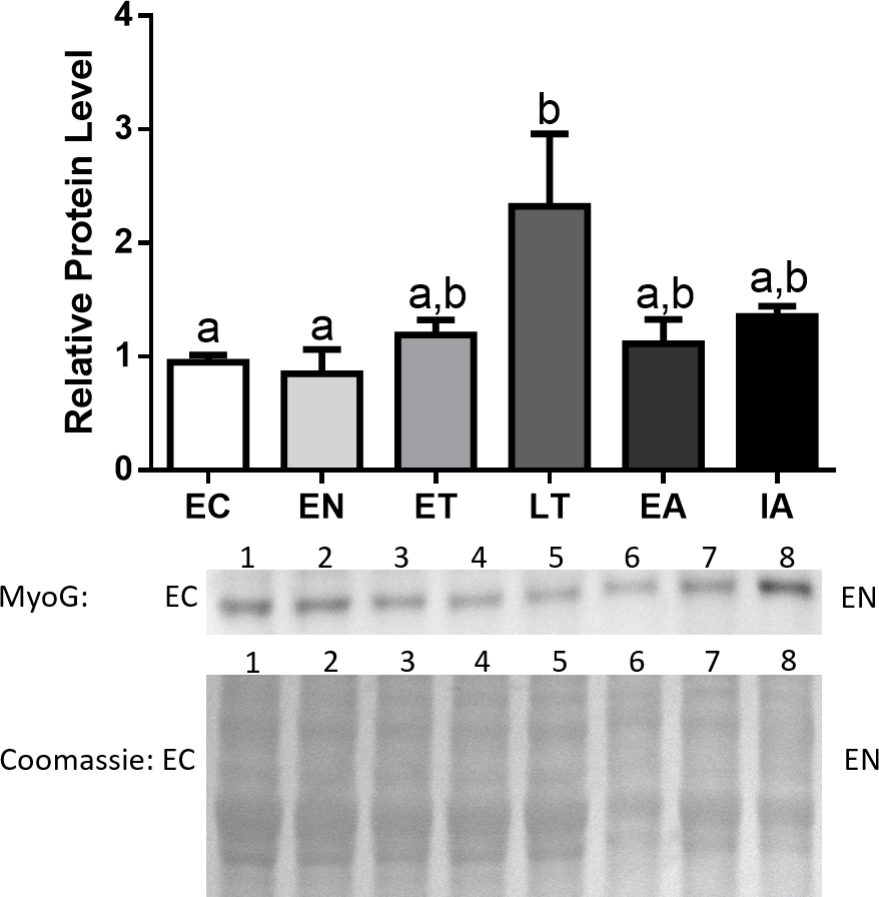
Changes in protein levels of MyoG over the course of the torpor-arousal cycle in the cardiac muscle of *I. tridecemlineatus*. Representative immunoblots and Coomassie total protein loading controls are shown for selected pairs of sampling points that are labeled on the left and right of the gel. Sample numbers (lanes) are labeled along the top indicating an *n* = 4 of one sampling point and an *n* = 4 of the other sampling point (i.e., for MyoG: EC lanes 1, 2, 3, 4 and EN lanes 5, 6, 7, 8). Histograms show mean band densitometries (±S.E.M., *n* = 4 independent protein isolations from different animals). Data were analyzed using analysis of variance with a post hoc Tukey’s test (*p* < 0.05); for each parameter measured, values that are not statistically different from each other share the same letter notation.

MyoG protein levels remained constant throughout the torpor-arousal cycle with the exception of LT, where MyoG levels peaked and were significantly elevated by 2.4-fold in comparison with EC (Fig. 4).

**Figure 5:**
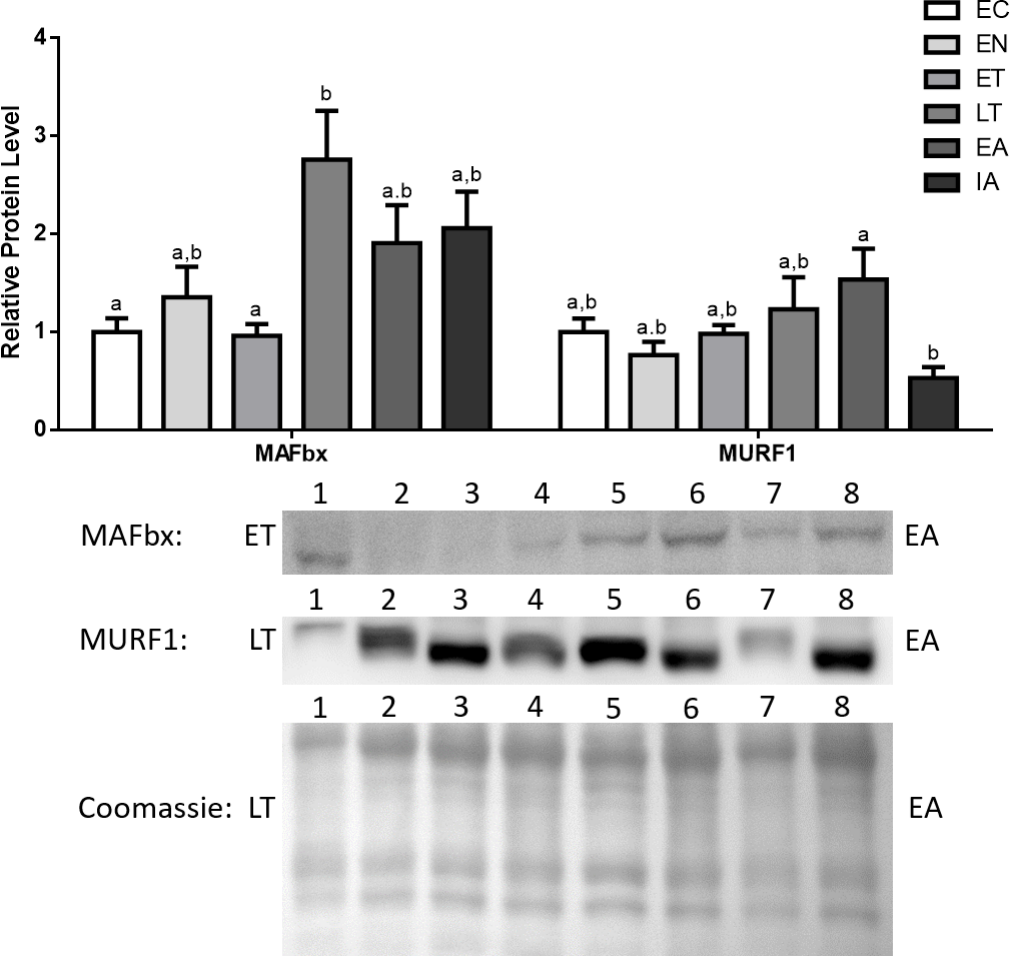
Changes in protein levels if the ubiquitin ligases MAFbx and MuRF1 over the course of the torpor-arousal cycle in the cardiac muscle of *I. tridecemlineatus*. Representative immunoblots and Coomassie total protein loading controls are shown for selected pairs of sampling points that are labeled on the left and right of the gel. Sample numbers (lanes) are labeled along the top indicating an *n* = 4 of one sampling point and an *n* = 4 of the other sampling point (i.e., for MAFbx: ET lanes 1, 2, 3, 4 and EA lanes 5, 6, 7, 8). Histograms show mean band densitometries (±S.E.M., *n* = 4 independent protein isolations from different animals). Data were analyzed using analysis of variance with a post hoc Tukey’s test (*p* < 0.05); for each parameter measured, values that are not statistically different from each other share the same letter notation.

MAFbx protein levels remained fairly stable during EN and ET, and then increased during LT (2.8-fold increase in comparison with EC). Afterwards, MAFbx levels remained elevated during EA and interbout arousal (IA) (1.9- and 2.1-fold increases relative to EC for EA and IA, respectively) (Fig. 5). MuRF1 protein levels also remained stable during EN and ET, then began increasing during LT and peaked during EA (1.23- and 1.54-fold increases in comparison with EC) (Fig. 5).

## Discussion

The present study furthers our understanding of the molecular mechanisms underlying cardiac muscle remodelling during hibernation in 13-lined ground squirrels (*I. tridecemlineatus*). Specifically, the study explores the regulatory mechanisms controlling cardiac muscle-expression of E3 ubiquitin ligases during hibernation. Western blotting results indicate that MAFbx and MuRF1 both showed significant increases during late torpor (LT) and early arousal (EA) by as much as 2.8-fold from euthermic control (EC) (Fig. 5). This indicates an activation of the E3 ubiquitin ligase system during LT and arousal, which in turn suggests that protein turnover and cardiac muscle remodeling could be occurring at later stages of the torpor-arousal cycle. These results support the idea of cardiac muscle plasticity in hibernating animals (Wickler, Hoyt & Van Breukelen, 1991; Nelson & Rourke, 2013). The elevated levels of MAFbx extended into interbout arousal (IA) while MuRF1 expression returned to euthermic levels during IA, when the squirrel was fully aroused and its *T*_*b*_ has returned to 37 °C. This suggests that upregulation of protein degradation by MAFbx and MuRF1 could occur briefly as the animal is being aroused. These findings match the results from a transcriptomics study done by Hampton et al. (2011), where it was observed that MAFbx mRNA levels peaked during torpor and remained elevated during IA, whereas MuRF1 levels were elevated during torpor as well but returned to euthermic levels during IA in comparison with animals in October (EC) (Hampton et al., 2011). Although further studies need to be conducted to detect whether observable changes in cardiac muscle dimensions or structure take place during torpor and arousal, it is likely that the tight control of MAFbx and MuRF1 expression acts as a mechanism to degrade unwanted proteins, whose accumulation may lead to cardiac fibrosis, for instance. In another hibernator, the hibernating grizzly bear, it was shown that both collagen type I and type III did not increase during hibernation (Nelson et al., 2008), and the UPS may play a role in this process.

Due to the role of transcription factors as central regulators of proteins that promote muscle atrophy, such as MAFbx and MuRF1, we analyzed the protein expression of numerous regulators of the E3 ligases, starting with MyoG. Protein expression of this transcription factor showed significant upregulation during LT, where MyoG increased 2.4-fold relative to EC (Fig. 4). This data suggests that upregulation of these two positive regulators of MAFbx and MuRF1 during LT could initiate the rise in MAFbx and MuRF1 protein levels during LT –possibly leading to protein degradation in the heart. Furthermore, MyoG levels increased during LT, which could be in response to activation of the NFAT-calcineurin pathway in ground squirrel cardiac muscle during torpor (Zhang & Storey, 2015). The NFAT-calcineurin pathway has been shown to regulate the expression of MyoG as NFATc2 and c3 are able to bind to the *myog* promoter (Armand et al., 2008). Since the NFAT-calcineurin pathway is an important mechanism that promotes cardiac hypertrophy during torpor, the upregulation of MyoG by NFAT during LT may act as a feedback mechanism in squirrel cardiac muscle to inhibit hypertrophy-promoting pathways. This negative feedback mechanism is supported by studies that show MAFbx ubiquitinates and targets calcineurin for degradation, thereby attenuating if not reversing cardiac hypertrophy in mouse models (Li et al., 2007; Baskin et al., 2014). The pattern of expression throughout the torpor-arousal cycle from this study seem to contradict the gene expression data from Hampton et al. (2011), as MyoG mRNA levels remain unchanged from euthermia to torpor, and to arousal. In contrast, the protein levels of MyoG increased significantly at LT, and one explanation is that the torpor samples collected by Hampton et al. (2011) may not be at the LT time point. Given the dramatic changes in in gene and protein expression over a short period of time during hibernation, comparisons between studies must be made when the conditions are kept the same.

In addition, the protein levels of total and phosphorylated Foxo1 and Foxo3a were also analyzed. It should be noted that Foxo1 and 3a showed increased expression and activity earlier in the torpor-arousal cycle than MyoG did (Figs. 2 and 4). Foxo1 protein levels peaked at EN (2.3-fold relative to EC) (Fig. 2). Foxo3a on the other hand showed very high levels throughout the torpor-arousal cycle with the exception of EC (Fig. 3). Furthermore, the Foxo1 amino acid residues Ser^319^ and Thr^24^ are sites that Akt can phosphorylate in order to prevent the nuclear translocation of Foxo1; thus preventing its regulation of downstream targets. Thr^32^ is an Akt phosphorylation site on Foxo3a that plays the identical role (Dobson et al., 2011). When we analyzed the levels of phosphorylated Foxo1 throughout torpor, levels of both phosphorylated forms showed at least a 45% decrease from EC (Fig. 2). For Foxo3a, levels of phosphorylated Thr^32^ decreased by at least 66% from EC throughout torpor. Another p-Foxo3a residue that was analyzed was Ser^318/321^, which is phosphorylated by casein kinase 1 (CK1) to promote nuclear exclusion of Foxo3a, levels of this residue increased during ET (2.85-fold relative to EN, *p* < 0.05) (Fig. 3). Therefore, we conclude that there was not only an increase in Foxo1 and Foxo3a levels during torpor, but there are also large elevation in Foxo1 and 3a activity, as defined by an increase in the amount of dephosphorylated or nuclear Foxo.

Again, there are some inconsistencies when comparing our protein level results to the transcriptomics/gene profiling results of previous studies (Hampton et al., 2011; Vermillion et al., 2015a). Foxo1 showed no changes during torpor and IA in either study, and Foxo3a no changes as well during the two above mentioned time points in the Hampton et al. (2011) study, but its mRNA levels actually decreased during torpor and IA compared to euthermia in the findings of Vermillion et al. (2015a). In the Vermillion et al. (2015a) study, torpor samples were collected after at least three days of torpor, but our LT samples were collected after at least five days. Therefore, for Foxo1, the same explanation for MyoG applies, because the earlier torpor time point could mean that gene expression was characterized between ET and LT, where our results show that Foxo1 levels are unchanged from EC. On the other hand, there is clearly a difference in our Foxo3a results, where protein levels are increased from euthermic levels throughout torpor, and those results of the two transcriptomics studies. The ground squirrel has many other unique mechanisms of regulating translation and protein synthesis while its metabolic rate is depressed during hibernation. Some of these mechanisms include selective losses of polysomes and storage of certain mRNAs in monosomes that confer increased mRNA stability to specific mRNA at various stages during the torpor-arousal cycle (Grabek et al., 2015). Therefore, Foxo3a mRNA may be one of the essential genes whose mRNA is stored in monosomes so that it can be translated quickly in order to transcriptionally regulate processes like cell cycle progression and antioxidant response. Also, the storage of mRNAs in stress granules could also serve to store essential mRNAs for genes like Foxo3a so that translation of the key transcripts can be rapidly initiated when the Foxo3a protein is needed throughout the torpor-arousal cycle.

These results are consistent with transcriptomics analysis on ground squirrels during hibernation, which show an inhibition of the IGF/PI3K/Akt pathway during torpor (Vermillion et al., 2015a), suggesting dephosphorylation and nuclear localization of the Foxo transcription factors. A reason for these increases in Foxo1 and 3a expression and activation could be related to antioxidant response. In ground squirrels, reactive oxygen species (ROS) accumulates during EN as well as arousal. This increase of ROS may be initiating the transcriptional regulation of targets involved in both protein degradation and antioxidant response through the Foxo transcription factors (Allan & Storey, 2012; Brown et al., 2012; Wu & Storey, 2014). This could explain the peak in Foxo1 levels at EN, and the increase in p-Foxo3a Ser^318/321^ levels during ET to promote nuclear exclusion and inactivation of Foxo3a after ROS levels have decreased. Although further research is required to further elucidate the specific roles of individual Foxo transcription factors at various stages of the torpor-arousal cycle, previous work has suggested that different Foxo proteins have different binding affinities to MAFbx and MuRF1 (Waddell et al., 2008). Results, from our study suggest that Foxo1 and 3a could both positively regulate the cardiac expression of MAFbx and MuRF1 in hibernating ground squirrels. However, MyoG may control the expression of MAFbx and MuRF1 to a greater extent in our animal that Foxo1 or 3a, given that the expression of the E3 ligases increase as MyoG levels increase during LT. One explanation for the differential regulation E3 ligases by the three transcription factors is that these transcription factors show differential binding of regulation of genes. For example, it has been observed that inhibiting MyoG through the use of trichostatin A results in reduced muscle wasting, and there is a greater decline in MAFbx expression in comparison with MuRF1 expression (Bricceno et al., 2012). This data suggests that MyoG may regulate the expression of MAFbx to a greater extent than MuRF1. In addition, it was also observed that not all Foxo family members bind equally to the promoters of MAFbx and MURF1. For instance, Foxo1 activates the Foxo binding motif; leading to MURF1 upregulation, to a greater degree than Foxo3a or 4 (Waddell et al., 2008). Also, miRNAs can regulate the translation of MAFbx and MuRF1 transcripts. For example, miR-23a suppresses the translation of both MAFbx and MuRF1 (Wada et al., 2011).

## Conclusion

The present study provides novel insights into some of the important proteins involved in cardiac muscle remodelling during hibernation. The results from this study indicate an upregulation of the E3 ligases MAFbx and MuRF1 in addition to their regulators, mainly MyoG, in a coordinated fashion during late torpor and arousal. The coordination of MyoG, MAFbx, and MuRF1 expression suggests that MyoG may be a specific regulator of adaptive responses in the heart to avoid pathological hypertrophy, whereas Foxo1 and 3a may primarily regulate other processes such as anti-oxidant response in the heart. Therefore, in our animals, the increase in expression of the ubiquitination machinery may occur during torpor, albeit late, in response to an increase in protein synthesis as hypertrophy-promoting pathways are activated earlier during torpor. These novel findings on this natural physiological process advances our knowledge of cardiac muscle remodeling and plasticity, and highlights the possibility of using this model to study the molecular mechanisms underlying adaptive or physiological cardiac hypertrophy.

## Additional Information and Declarations

### Data Availability

The following information was supplied regarding data availability: The research in this article did not generate any raw data.
